# Deep Learning–Assisted Gait Parameter Assessment for Neurodegenerative Diseases: Model Development and Validation

**DOI:** 10.2196/46427

**Published:** 2023-07-05

**Authors:** Yu Jing, Peinuan Qin, Xiangmin Fan, Wei Qiang, Zhu Wencheng, Wei Sun, Feng Tian, Dakuo Wang

**Affiliations:** 1 Institute of Software, Chinese Academy of Sciences Beijing China; 2 University of Chinese Academy of Sciences Beijing China; 3 The University of Melbourne Melbourne Australia; 4 Chinese Academy of Sciences - Ruiyi Beijing China; 5 IBM Research Cambridge, MA United States

**Keywords:** deep learning, neurodegenerative disease, auxiliary medical care, gait parameter assessment

## Abstract

**Background:**

Neurodegenerative diseases (NDDs) are prevalent among older adults worldwide. Early diagnosis of NDD is challenging yet crucial. Gait status has been identified as an indicator of early-stage NDD changes and can play a significant role in diagnosis, treatment, and rehabilitation. Historically, gait assessment has relied on intricate but imprecise scales by trained professionals or required patients to wear additional equipment, causing discomfort. Advancements in artificial intelligence may completely transform this and offer a novel approach to gait evaluation.

**Objective:**

This study aimed to use cutting-edge machine learning techniques to offer patients a noninvasive, entirely contactless gait assessment and provide health care professionals with precise gait assessment results covering all common gait-related parameters to assist in diagnosis and rehabilitation planning.

**Methods:**

Data collection involved motion data from 41 different participants aged 25 to 85 (mean 57.51, SD 12.93) years captured in motion sequences using the Azure Kinect (Microsoft Corp; a 3D camera with a 30-Hz sampling frequency). Support vector machine (SVM) and bidirectional long short-term memory (Bi-LSTM) classifiers trained using spatiotemporal features extracted from raw data were used to identify gait types in each walking frame. Gait semantics could then be obtained from the frame labels, and all the gait parameters could be calculated accordingly. For optimal generalization performance of the model, the classifiers were trained using a 10-fold cross-validation strategy. The proposed algorithm was also compared with the previous best heuristic method. Qualitative and quantitative feedback from medical staff and patients in actual medical scenarios was extensively collected for usability analysis.

**Results:**

The evaluations comprised 3 aspects. Regarding the classification results from the 2 classifiers, Bi-LSTM achieved an average precision, recall, and *F*_1_-score of 90.54%, 90.41%, and 90.38%, respectively, whereas these metrics were 86.99%, 86.62%, and 86.67%, respectively, for SVM. Moreover, the Bi-LSTM–based method attained 93.2% accuracy in gait segmentation evaluation (tolerance set to 2), whereas that of the SVM-based method achieved only 77.5% accuracy. For the final gait parameter calculation result, the average error rate of the heuristic method, SVM, and Bi-LSTM was 20.91% (SD 24.69%), 5.85% (SD 5.45%), and 3.17% (SD 2.75%), respectively.

**Conclusions:**

This study demonstrated that the Bi-LSTM–based approach can effectively support accurate gait parameter assessment, assisting medical professionals in making early diagnoses and reasonable rehabilitation plans for patients with NDD.

## Introduction

### Background

Neurodegenerative diseases (NDDs) have an insidious onset, long duration, and limited effective treatment options and primarily affect middle-aged and older individuals. NDDs also result in substantial economic losses annually. A survey in Sweden [1] reported that the total cost of developing Parkinson disease per person was at €13,800 (US $14,763.50) per year. A study by Yang et al [2] revealed a US prevalence of approximately 1 million individuals diagnosed with Parkinson disease in 2017 and a total economic burden of US $51.9 billion. Gait change, present in the early stages of nearly all NDDs [3], could play a crucial role in early diagnosis if accurately assessed. Early diagnosis allows potential patients to modify their lifestyle, seek active medication, and slow disease progression, thus improving their quality of life [4,5].

Current NDD diagnosis predominantly relies on standardized scales [6-9], which exhibit considerable error. The quality of patient-physician communication and manual scale recording can affect disease quantification. It has been demonstrated that, even for specialist neuroscientists focusing on movement disorders, diagnostic accuracy error rates have reached approximately 20% [10]. For instance, the Unified Parkinson’s Disease Rating Scale [6] has been widely used because of its simplicity and established clinical validation. These methods generally rely on clinicians’ expertise to assess patients’ symptoms and assign scores accordingly. However, these scales are subject to interrater variability and potential observer bias and can be influenced by physicians’ subjective experiences. Although widely adopted, these limitations highlight the need for more accurate, objective, and reliable assessment methods that can reduce human error and improve the diagnostic process for NDDs.

To satisfy this need, numerous studies are exploring progressive sensors or advanced artificial intelligence algorithms as auxiliary assessment methods, which can be divided into contact and noncontact approaches. Contact methods use gait-related tasks using wearable-based inertial measurement unit sensors [11] or optical matching with marker points on the human body [12], resulting in intrusiveness [13-15] and potentially preventing patients from exhibiting their problems in a relaxed state. Noncontact methods, relying on computer vision [16-19] or signals [20], improve this situation. Therefore, we chose Kinect (Microsoft Corp) [21] for data collection because of its noninvasive nature, real-time data capture capability, cost-effectiveness, and ability to capture rich 3D skeletal data. These advantages make Kinect a suitable choice for gait analysis in both clinical and research settings.

Before this study, the most common approach to achieving this objective involved heuristic methods [22-25]. Latorre et al [26] compared various heuristic methods, finding that using ankle speed to locate the heel strike and toe-off and obtain the gait parameter could yield the highest accuracy for gait parameter calculation among heuristic methods. Despite their simplicity, these methods have not been widely recognized by physicians owing to their unsatisfactory assessment performance. In our opinion, they are flawed in at least 3 aspects (Textbox 1).

Considering these limitations, there is a clear need for a more comprehensive and accurate approach to gait assessment that addresses these shortcomings and better supports the early diagnosis of NDDs.

Inspired by these drawbacks, we developed our method by combining multiple spatiotemporal features, carefully constructing gait cycles, and leveraging timing information. Simultaneously, we aimed to use machine learning advantages to overcome the heuristic weaknesses. To the best of our knowledge, our approach is the first to use machine learning to obtain accurate gait parameter results based on the precise gait semantic segmentation. Specifically, we initially used Kinect to capture the raw skeleton information and designed 32 spatiotemporal features accordingly. We then pioneered a 2-stage approach to gait assessment. The first stage combined features to train classifiers for the gait states of each frame. The second stage extracted the gait semantics based on predicted gait states and obtained the legitimate sequence of gait semantics by filtering gait semantics. The legitimate sequence of gait semantics enabled the further calculation of accurate gait-related parameters.

**Flaws of the heuristic methods.**

**Overreliance on a single fixed feature**

- Heuristic methods often depend on just 1 feature for gait analysis, which may not capture the complexity of gait patterns in patients with neurodegenerative diseases. This reliance on a single feature makes it challenging to assess the overall gait performance accurately.

**Inability to achieve precise segmentation of gait semantics**

- Heuristic methods often struggle to segment gait cycles accurately, which is crucial for understanding the subtle changes in gait patterns associated with neurodegenerative diseases. This limitation can lead to inaccuracies in the assessment of gait performance and hinder early diagnosis.

**Underuse of timing information**

- Heuristic methods do not make full use of the timing information present in gait data. Incorporating temporal features is essential for understanding the dynamics of gait patterns and detecting potential abnormalities. By neglecting the temporal aspect, these methods may miss critical information that could improve the accuracy of gait assessment.

### Objectives

In this study, we primarily focused on validating the effectiveness of our proposed noncontact gait analysis method using Kinect technology and machine learning for the early diagnosis of NDDs. In addition, we aimed to assess the usability of our method to ensure that it can be easily adopted in clinical settings for more accurate and efficient gait analysis. By addressing both validation and usability aspects, our research aimed to offer a comprehensive solution for the early diagnosis of NDDs, which can potentially improve the quality of life of affected individuals.

## Methods

### Overview

**Figure 1:**
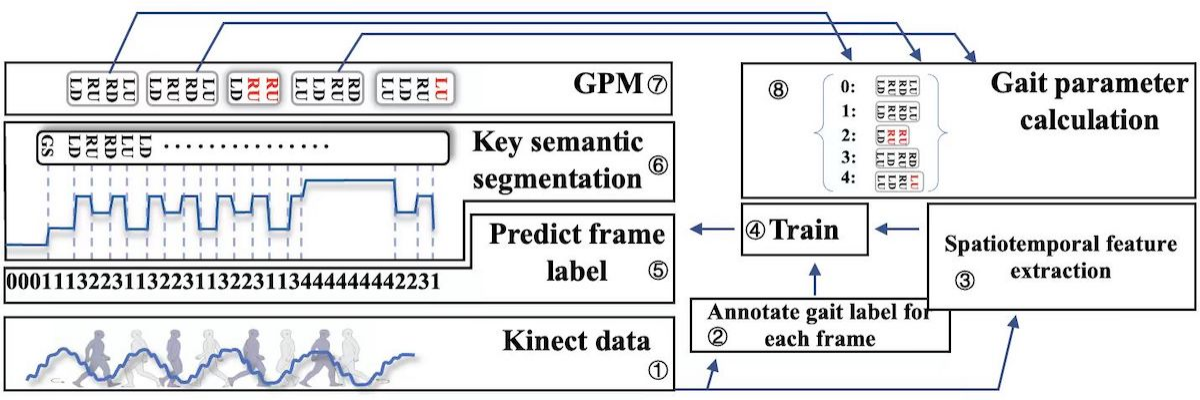
The overview of our algorithm. LD: left foot down; LU: left foot up; GPM: gait pair mechanism; RD: right foot down; RU: right foot up.

The overview of our method is illustrated in Figure 1, with steps 1 to 3 representing the preprocessing stage, involving data collection and labeling. Step 4 involved training the frame-to-frame classifier, whereas steps 5 to 7 involved selecting the appropriate gait cycles. Finally, in step 8, the parameters were calculated based on the outcome of step 7.

### Recruitment

A total of 41 participants were recruited from a hospital: 30 (73%) male and 11 (27%) female participants. Data collection was conducted safely under the direction and supervision of physicians in compliance with the local ethics policy, with informed consent obtained from all participants. All data collected in this study were secondary analyses of previously collected research data, and the original informed consent obtained during the primary data collection process included provisions for secondary analysis without the need for additional consent. Participants’ privacy and confidentiality were protected throughout the study. All collected data were deidentified, and any potentially identifying information was removed before analysis. Measures were taken to ensure that the data were stored securely, and access to the data was limited to authorized members of the research team. The age range of the data set was 25 to 85 years, and the average age was 57.51 (SD 12.93) years. The age distribution is shown in Table 1. By studying a sample with a wide age distribution, we could better assess the applicability and accuracy of our approach in patients of different ages. More specific statistical values of actual gait parameters in the data set are shown in Table 2. The distribution of the data set was differentiated; thus, it was feasible to take this data set into use for our training and evaluation.

**Table 1:** Age distribution of the participants (N=41).

Age range (years)	Participants, n (%)
25-35	2 (5)
35-45	5 (12)
45-55	6 (15)
55-65	16 (39)
65-75	11 (27)
75-85	1 (2)

**Table 2:** Statistics of the actual parameters of the data set.

Gait parameter (unit)	Minimum	First quartile	Median	Third quartile	Maximum
Speed (m/s)	0.540	0.895	0.979	1.046	1.428
LeftStride (m)	0.497	0.926	1.025	1.129	1.437
RightStride (m)	0.498	0.943	1.024	1.113	1.334
LeftStrideSpeed (m/s)	0.510	0.947	1.036	1.186	1.677
RightStrideSpeed (m/s)	0.521	0.935	1.049	1.169	1.483
LeftStep (m)	0.163	0.464	0.527	0.571	0.693
RightStep (m)	0.278	0.437	0.493	0.523	0.706
LeftCadence (steps/min)	89.570	112.264	125.067	134.620	154.677
RightCadence (steps/min)	87.901	116.061	125.974	133.074	143.690
LeftCycle (s)	0.812	0.895	0.967	1.039	1.352
RightCycle (s)	0.808	0.894	0.956	1.067	1.333
LeftSwing (%)	20.910	27.203	29.378	30.363	37.958
RightSwing (%)	22.850	27.631	29.628	31.464	35.674
LeftStance (%)	62.042	69.637	70.622	72.797	79.090
RightStance (%)	64.326	68.536	70.372	72.369	77.150
DoubleSupport (%)	27.825	39.076	40.867	43.992	54.593
LeftSwingSpeed (m/s)	1.495	2.361	2.555	2.923	3.519
RightSwingSpeed (m/s)	1.598	2.236	2.534	2.830	3.589

### Procedure

**Figure 2:**
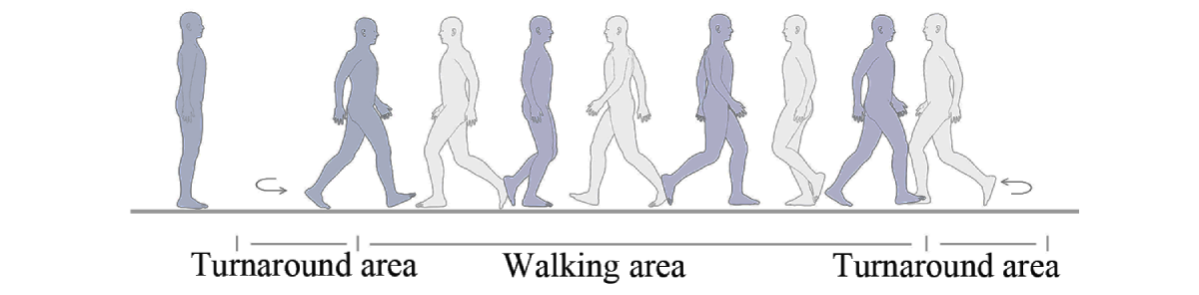
A decomposition of gait states during a walking round trip.

We used the Azure Kinect [17], a 3D camera developed by Microsoft, positioned 0.65 m from the ground to record the gait data. A walking area of 5 m in length was established in front of the Kinect. Participants were asked to walk back and forth 3 times in a natural walking posture, including actions of standing still, moving forward, and turning around, as shown in Figure 2. Data collection started when the participant was standing still at the starting point and stopped when they completed the final turnaround and stood still again. The length of the video depended on the walking status of each participant, typically taking approximately 20 seconds for participants without NDDs and longer for those exhibiting gait patterns associated with NDDs. After data collection, each frame of the video was annotated using an open-source annotation tool [27].

### Data Annotation

#### Overview

To label each frame in the data sequence, we defined 5 types of labels and provided an explanation for their use. We also introduced the concept of gait semantics and described how to precisely locate them from labeled frames. On the basis of these semantics, we were able to calculate the gait parameters.

#### Label Definition

**Figure 3:**
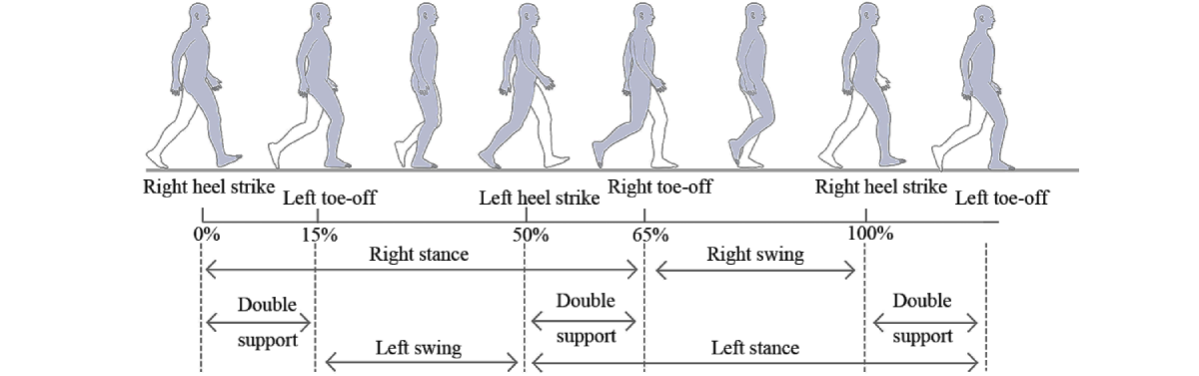
The division of gait states during straight ahead.

We defined 5 states for a complete gait cycle: standstill(S), left swing(L), double support(D), right swing(R), and turnaround(T) (as shown in Figure 3). This was done because a gait cycle is a dynamic process that combines both feet’s movements. If we only focus on the left or right foot, a gait cycle consists of left foot swing, left foot support, right foot swing, and right foot support. However, there is a brief cross when the left and right foot move continuously, when both feet are in the landing state, that is different from a standstill. Medically, this phenomenon is referred to as *double support*.

#### Gait Semantic Definition

**Figure 4:**
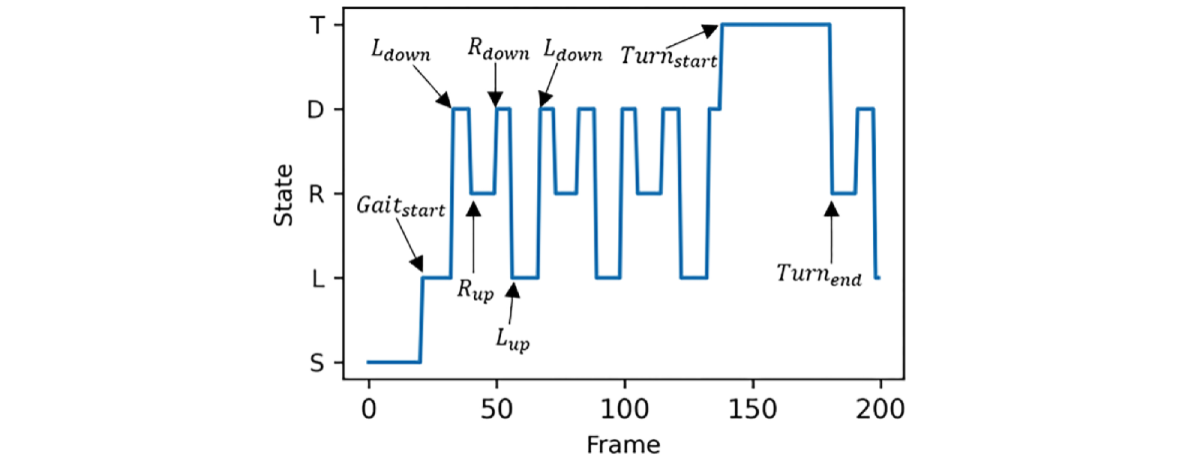
Schematic diagram of the state of the gait process. Ldown: left foot down; Lup: left foot up; Rdown: right foot down; Rup: right foot up; Turnstart: turnaround start; Turnend: turnaround end.

We visualized the labels of a piece of gait data, with each frame corresponding to a unique label to mark the current gait state (shown in Figure 4). We defined 7 gait semantics that served as localizers for state transitions of gait: gait start (*Gait_start_*), left foot up (*L_up_*), left foot down (*L_down_*), right foot up (*R_up_*), right foot down (*R_down_*), turnaround start (*Turn_start_*), and turnaround end (*Turn_end_*). These semantics were obtained by slicing the successive gait states and calculated as per equations 1 to 7. Precise localization of these semantics is crucial for calculating the final gait parameters.

*L_down_*: (*state_i_*=*D*) ∧ (*state_i_*_− 1_=*L*) **(1)**

*_Rdown_*: (*state_i_*=*D*) ∧ (*state_i_*_− 1_=*R*) **(2)**

*L_up_*: (*state_i_*=*L*) ∧ (*state_i_*_− 1_=*D*) **(3)**

*R_up_*: (*state_i_*=*R*) ∧ (*state_i_*_− 1_=*D*) **(4)**

*Turn_start_*: (*state_i_*=*T*) ∧ (*state_i_*_− 1_≠*T*) **(5)**

*Turn_end_*: (*state_i_≠T*) ∧ (*state_i_*_− 1_=*T*) **(6)**

*Gait_start_*: (*state_i_*≠*S*) ∧ (*state_i_*_− 1_=*S*) **(7)**

In these equations, *state_i_* and *state_i_*_− 1_ indicate the gait state of the *i*th and (*i* − 1)th frame, respectively.

### Feature Design and Extraction

We designed features that considered spatiotemporal motion and relative position between skeletal points. The lower body consists of 9 pivotal points: the “pelvis,” “left hip,” “right hip,” “left knee,” “right knee,” “left ankle,” “right ankle,” “left foot,” and “right foot.” The temporal feature contains the speed of these points in the 3 directions (*x*, *y*, and *z*). The 32 features listed in Textbox 2 constitute the overall feature tensor of 1 data frame.

**Spatial, temporal, and movement features used in our method.**

**Temporal**

- Joint speed (3 × lower body joint [*x*, *y*, and *z*])

**Spatial**

- Distance between people and the camera (1)

**Movement**

- Distance between the knees (1)
- Distance between the pelvis and the feet (2)
- Angle of waist rotation (1)

### Algorithm

#### Overview

Our algorithm consists of 2 stages. In the first stage, we constructed a classifier for single-frame gait label prediction based on the features (as mentioned in the Feature Design and Extraction section). We explored a non–time-series classifier (support vector machine [SVM] [28]) and a time-series classifier (bidirectional long short-term memory [Bi-LSTM] [29]) to determine the most suitable option for the current application scenario. In the second phase, gait semantics were identified and located, and available gait cycles were selected using our proposed gait pair mechanism (GPM; detailed description in the *Segmentation, GPM, and Calculation* section) to calculate precise gait parameters.

#### Preprocessing

Each gait label was associated with a single frame, whereas the Azure Kinect captured data at a rate of 30 frames per second, yielding 30 gait labels per second. In SVM, individual frames served as inputs for training and prediction processes. Conversely, for the Bi-LSTM, motion sequences were input into the model maintaining a fixed sequence length (*seq_len*=5 in practice). Subsequently, each participant’s gait process was partitioned into sequences, which were supplemented with integer 0 in instances where the sequences were insufficient in length. Following randomization of all sequences, they were introduced into the Bi-LSTM classifier to facilitate the training process.

#### Frame Classification

For the first-stage classifier selection, we evaluated both the non–time-series SVM and the time-series Bi-LSTM classifiers.

##### Non–Time-Series Classification

We used the SVM classifier with a radial basis function kernel as the representative for non–time-series classifiers for the reasons outlined in Textbox 3.

In summary, we chose SVM as a nonserial model because of its strong generalization ability, high flexibility, and successful application to real-world problems. These characteristics make SVM a suitable nonserial model for this study.

**Reasons for using support vector machine (SVM) as the representative for non–time-series classifiers.**

**SVMs have a strong generalization capability [28]**

- SVMs provide better generalization performance based on the principle of structural risk minimization. This means that SVMs can produce relatively good results when the model is applied to new, unseen data.

**SVMs are highly flexible [30]**

- SVMs can use different kernel functions to handle both linear and nonlinear problems. This allows SVMs to adapt to various data distributions and provide better classification performance.

**Successful applications of SVMs to real-world problems [31]**

- SVM has demonstrated its superior performance in many real-world problems in areas such as image classification and text classification. This shows that SVM has high practicality and can be well adapted to various problems.

##### Time-Series Classification

We used Bi-LSTM as the representative for time-series classifiers for the reasons outlined in Textbox 4.

**Reasons for using bidirectional long short-term memory (Bi-LSTM) as the representative for time-series classifiers.**

**Long short-term memory (LSTM) [32]**

- LSTM is a special type of recurrent neural network (RNN) that captures long-term dependencies and solves the gradient disappearance or gradient explosion problem faced by traditional RNNs when dealing with long sequences. LSTM has been shown to have superior performance in many time-series tasks, especially when dealing with sequential data with complex dependencies.

**Bidirectional model**

- Bi-LSTM is a variant of LSTM that captures contextual relationships in sequences by learning both forward and backward information. This allows Bi-LSTM to better understand the patterns and dependencies in a gait sequence, ultimately improving the prediction of gait labels for each frame [33].

**Maturity and wide application**

- Bi-LSTM has achieved remarkable results in many fields (eg, speech recognition [33] and medicine [34]), demonstrating its capability in processing time-series data. Moreover, as a large amount of experience has been accumulated on Bi-LSTM in previous research, there are many resources to draw from for implementation and optimization.

Although we are aware that other temporal models (eg, gated recurrent unit [35], 1D convolutional neural network [36], and transformer [37]) may have advantages in some aspects, based on a combination of performance, implementation difficulty, and existing experience, we believe that Bi-LSTM is an appropriate choice.

In choosing the classifiers for this study, we considered several factors, such as the effectiveness in capturing the complex patterns in gait analysis, computational complexity, and ease of deployment. First, by comparing different types of classifiers, we can gain a more comprehensive understanding of their strengths and limitations when tackling real-world gait analysis problems. This will help us provide valuable insights for future research and provide references for other researchers in the field of gait analysis. Second, although Bi-LSTM may outperform SVM in some aspects, we still need to demonstrate this empirically. In fact, SVM may have a better performance in some cases. For example, SVM has better robustness and effectiveness in handling high-dimensional data with lower generalization errors. Therefore, using SVM as a control group can help us verify the practical advantages of Bi-LSTM in this application area.

#### Segmentation, GPM, and Calculation

##### Overview

Obtaining precise gait semantic locations is essential for accurate parameter calculations. However, it is not enough; it is required that the gait semantics used for the calculations are correct. In more detail, if 2 consecutive gait states are incorrect owing to noise in the data, then the gait semantics generated by these gait states must be wrong even if they are extremely precise. Unfortunately, noise in the data is inevitable, so the aforementioned scenario is very likely to occur. To avoid the catastrophic consequences for the calculation results, it requires filtering out the subset that can be used for calculation from the known gait semantics. Hence, in this section, we propose an inspection method called GPM to ensure that all the gait cycles used for calculation are available.

##### Gait Segmentation

The first step is to locate the gait semantics from the labels predicted by the classifier.

##### Available Gait Cycles

**Figure 5:**
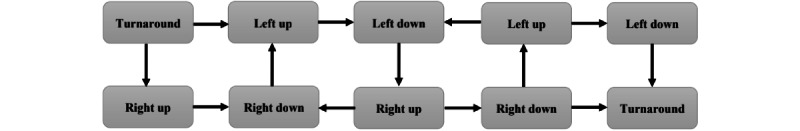
The legitimate sequence of gait semantics. The gray squares represent specific gait semantics. Starting from any gray square, the subsequent gait semantics defined in the direction of the arrow are legal.

The smallest unit we used to calculate gait parameters was the gait cycle, and to ensure the accuracy of the calculation, data located in the turnaround interval were ignored. This means that we only took data from straight intervals to construct gait cycles. Each complete and correct gait cycle should be composed of 4 consecutive gait semantics, for example, *L_up_* to *L_down_* to *R_up_* to *R_down_*. The starting position of this chain could be any semantic, and we list more examples of available gait chains in Figure 5. GPM is responsible for ensuring that only the correct gait cycle is retained. For instance, if a gait cycle contains an incorrect gait semantic sequence such as *L_up_* to *R_down_*, then the gait cycle will be discarded directly. Finally, after traversing the entire data sequence, we obtained all the available gait cycles in this data sequence, which were used for parameter calculation.

Some parameters must be calculated based on adjacent gait cycles (eg, equation 8), but they may have no neighbor (the GPM discards adjacent gait cycles). Therefore, we built a gait cycle dictionary for each data sequence (shown in Table 3) during the GPM operations to record. The key to the dictionary is a number that increases from 0, and the value is a gait cycle linked list.

**Table 3:** The dictionary built for maintaining the relative relationship between gait cycles during the gait pair mechanism.

Key	Value	Available
0	*L*_*down*_^a^ to *R*_*up*_^b^ to *R*_*down*_^c^ to *L*_*up*_^d^	Yes
1	*L*_*down*_ to *R*_*up*_ to *R*_*down*_ to *L*_*up*_	Yes
2	*L*_*down*_ to *R*_*up*_ to *R*_*up*_	No
3	*L*_*up*_ to *L*_*down*_ to *R*_*up*_ to *R*_*down*_	Yes
4	*L*_*up*_ to *L*_*down*_ to *R*_*up*_ to *L*_*up*_	No

^a^*L*_*down*_: left foot down

^b^*R*_*up*_: right foot up

^c^*R*_*down*_: right foot down

^d^*L*_*up*_: right foot up

##### Parameter Calculation

The parameters we calculated referred to gait standard parameters [38], which are shown in Table 4 in detail. After obtaining the available gait cycles through GPM, in each cycle, we can calculate swing time, height, and swing speed. In 2 identical cycles of the interval, we can obtain stride length, stride speed, and cycle time. Similarly, in the left and right cycles of the interval, we can obtain step length, double support time, and cadence. In addition, it is possible to determine the proportion of swing time, stance time, and double support time in the cycle using cycle time. We formulated the calculation of all parameters related to the left foot (equations 8-16), and the situation of the right foot was completely symmetrical.

Practically, the first and last gait semantics are not contained in any gait cycle as the gait is unstable at the start and end locations. Moreover, to make the calculation result reflect the condition of the gait process, we took the mean value of each parameter as the final gait parameter result. We obtained the walking speed by dividing the distance between the start and end points by the total time.

In these equations, *G^(i)^* represents the gait cycle with *key*=*i* in the dictionary (shown in Table 3), and distance(⋅) is the Euclidean distance between 2 ankles. *T*(⋅) is the method that obtains the time of the given gait semantic.




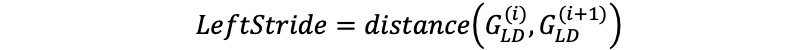

**(8)**






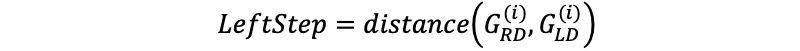

**(9)**






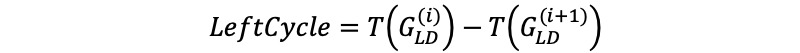

**(10)**





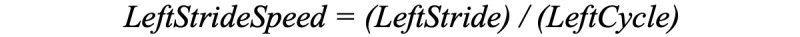

**(11)**





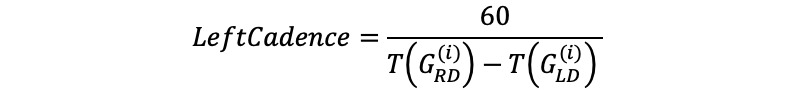

**(12)**






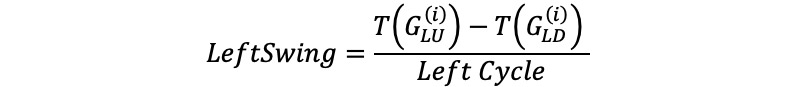

**(13)**






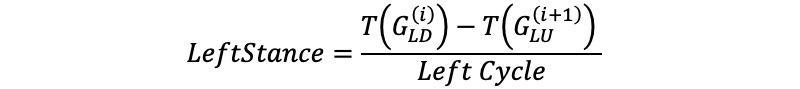

**(14)**






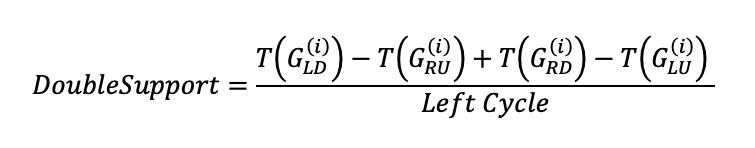

**(15)**






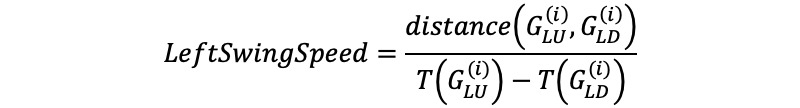

**(16)**



**Table 4:** The definition of all gait parameters that we intended to calculate.

Parameter (unit)	Definition
Speed (m/s)	Average speed during straight travel
LeftStride (m)	The distance between the 2 landings of the left foot
RightStride (m)	The distance between the 2 landings of the right foot
LeftStrideSpeed (m/s)	Average speed during a left stride
RightStrideSpeed (m/s)	Average speed during a right stride
LeftStep (m)	The distance between the landing of the left foot and the last landing of the right foot
RightStep (m)	The distance between the landing of the right foot and the last landing of the left foot
LeftCadence (steps per min)	Frequency of left footstep
RightCadence (steps per min)	Frequency of right footstep
LeftCycle (s)	Left stride cycle time
RightCycle (s)	Right stride cycle time
LeftSwing (%)	Percentage of swing phase time in the left stride period
RightSwing (%)	Percentage of swing phase time in the right stride period
LeftStance (%)	Percentage of stance phase time in the left stride period
RightStance (%)	Percentage of stance phase time in the right stride period
DoubleSupport (%)	Percentage of double support phase time in stride period
LeftSwingSpeed (m/s)	Average speed during a left swing
RightSwingSpeed (m/s)	Average speed during a right swing

### Evaluation

The evaluation covers 3 aspects: verifying the effectiveness of the classifiers, gait semantic segmentation, and gait parameter calculation.

#### 10-Fold Cross-Validation

This is a commonly used and effective method for model evaluation. All the experiments in this study were conducted through 10-fold cross-validation. Specifically, it is conducted by dividing the data set into 10 parts of the same size and each time selecting 1 part as the test set (different from all the previous test sets) and the rest as the training set, thereby training and evaluating an independent model. In total, 10 independent model evaluations were averaged to reflect the overall performance of the model.

#### Classification Evaluation

For the classification task, the common evaluation indicators are precision, recall, and *F*_1_-score (equations 17-20).




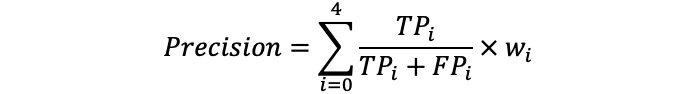


**(17)**




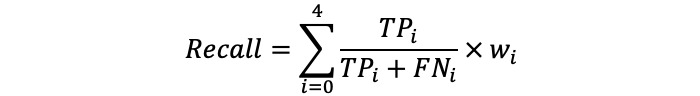


**(18)**




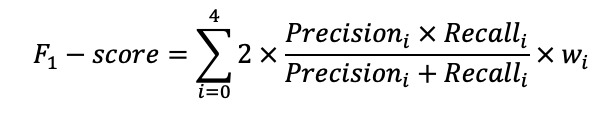


**(19)**




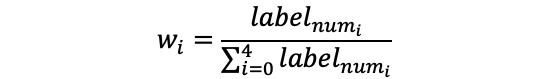


**(20)**

In these equations, *i* indicates that the current *i*th class is considered positive. TP, TN, FP, and FN denote of true positive, true negative, false positive, and false negative, respectively, where true or false means whether the predicted label is consistent with the ground truth and positive or negative indicates whether the current category is the class of interest. In the multiclassification problem, when a category is regarded as positive, all the other categories are regarded as negative. After calculating the precision and recall of each category, the final precision, recall, and *F*_1_-score were weighted based on the sample number of each category.

#### Gait Segmentation Evaluation

Let us assume that we obtain *K* gait semantics in a gait data sequence and this data sequence contains *n* frames in total. If the *k*th semantic point is located on the *j*th frame according to our algorithm but the ground truth location is the *j*th frame, then a hyperparameter *t* (representing the tolerance degree of location bias) is introduced to determine whether we would regard this semantic point as a mistake or an acceptable bias.




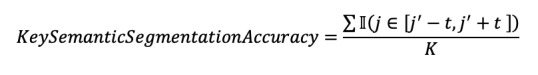


**(21)**

In these equations, 𝟙(⋅) denotes the indicator function, which means that, if *j* is in the range of (*j’* − *t, j’* + *t*), its value would be equal to 1; otherwise, it would be equal to 0.

#### Gait Parameter Calculation Evaluation

If we needed to calculate a total of *N* gait parameters, then, for the *i*th parameter to be calculated, we would use *V_p_^(i)^* to represent the value finally calculated by our algorithm. We could also figure out its corresponding precise value *V_g_^(i)^* using the ground truth. After that, depending on *V_p_^(i)^* and *V_g_^(i)^*, we could estimate the mean error and mean SD of all *N* gait parameters of these participants (equations 22 and 23).




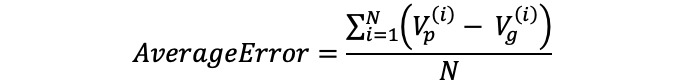


**(22)**




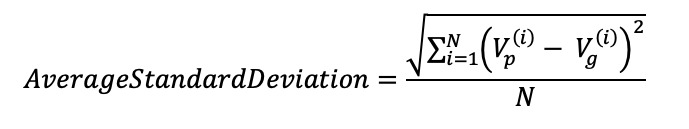


**(23)**

### Feedback Collection

#### Subjective Feedback

To verify the feasibility of this study’s method in field medical scenarios, we conducted in-depth interviews with a diverse group of participants, including 15 patients diagnosed with specific NDDs at various stages, 5 family members of the patients, and 5 health care workers experienced in NDD diagnosis. During the interviews, we collected a total of 6 conversation records from patients and their family members as well as 4 conversation records from health care workers. The interviews aimed to gather their insights and opinions on the acceptance of and preference for our proposed method compared with the traditional scale-based method, focusing on their subjective perceptions, experiences, and expectations in terms of usability, comfort, and overall satisfaction.

#### Quantitative Feedback

We developed a comprehensive evaluation system comprising several quantitative metrics: accuracy, convenience, practicability, invasiveness, and reliability. Each metric was scored on a scale ranging from 1 to 5, allowing for a detailed comparison between our proposed method and the traditional scale-based method. For this evaluation, patients were responsible for providing scores for convenience and invasiveness, reflecting their personal experience with each method. Meanwhile, physicians assessed accuracy, practicability, and reliability, offering a professional perspective on the effectiveness of each method in a clinical setting.

The patients involved in our quantitative and qualitative analyses were treated at the neurology departments of class 3A hospitals with which we collaborate. The physicians participating in our quantitative and qualitative analyses were practicing neurologists at these class 3A hospitals. We randomly selected 100 anonymous participants from multiple hospitals to complete this questionnaire, with a mix of 7 (7%) physicians and 93 (93%) patients, ensuring a diverse and representative sample.

### Ethics Approval

The Medical Review Ethics Committee of Peking Union Medical College Hospital approved the study (reference HS-3076).

## Results

### Classification and Gait Segmentation

**Figure 6:**
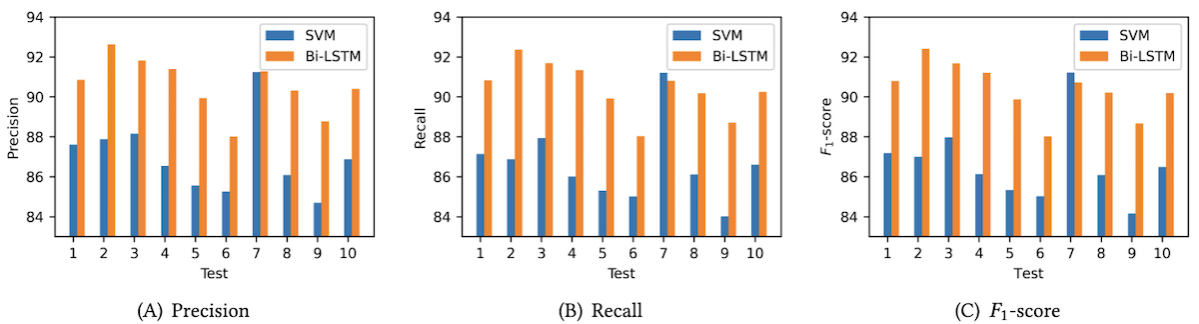
Performance comparison between the bidirectional long short-term memory (Bi-LSTM) and support vector machine (SVM) classifiers. (A) Precision; (B) Recall; (C) *F*_1_-score.

**Figure 7:**
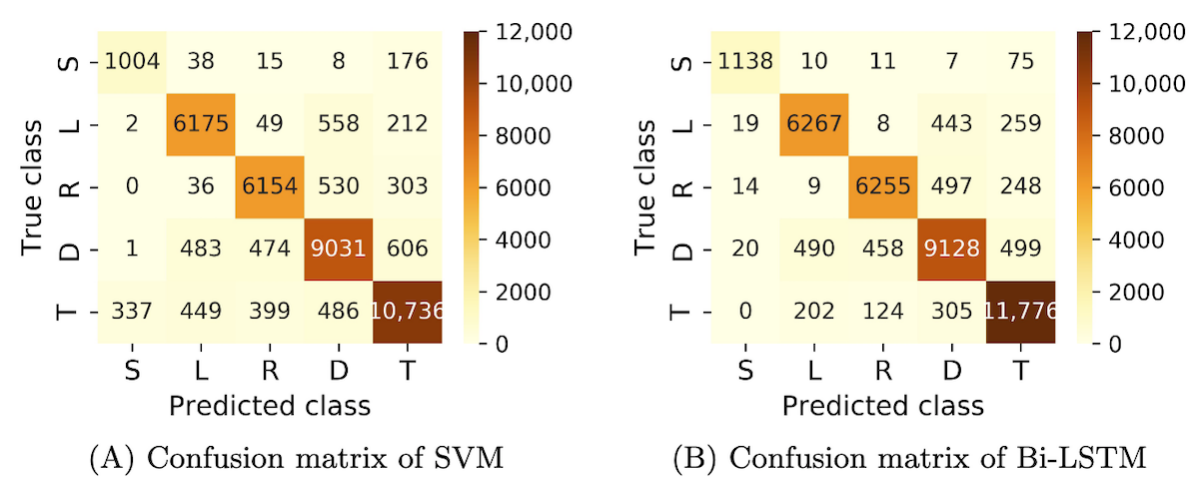
(A) Confusion matrix of support vector machine (SVM); (B) Confusion matrix of bidirectional long short-term memory (Bi-LSTM).

The comparison of the results of the 2 classifiers is demonstrated in Figure 6. The horizontal axis represents the number of cross-validations, and the vertical axis indicates the corresponding metric values (precision, recall, and *F*_1_-score). In 10 tests, the Bi-LSTM model predicted labels with an average precision of 90.54%, average recall of 90.41%, and average *F*_1_-score of 90.38%. In contrast, SVM yielded results of 86.99%, 86.62%, and 86.67% for precision, recall, and *F*_1_-score, respectively. The detailed confusion matrix for SVM and Bi-LSTM in the experiment is shown in Figures 7A and 7B. Moreover, the Bi-LSTM achieved an accuracy of 93.2% in gait segmentation evaluation (described in the *Gait Segmentation Evaluation* section, with the default tolerance set to 2), whereas the SVM only reached 77.5%.

### Gait Parameter Calculation Results

**Figure 8:**
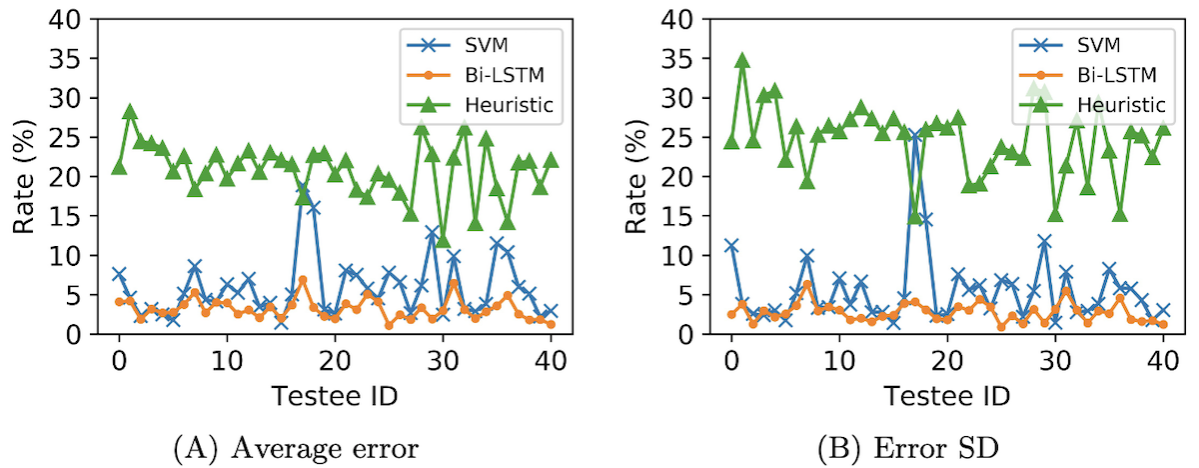
Parameter calculation errors for the heuristic method, support vector machine (SVM), and bidirectional long short-term memory (Bi-LSTM). (A) Average error; (B) Error SD.

As the heuristic method proposed in the study by Latorre et al [26] demonstrates the best parameter calculation, in this section, we compare it with our proposed methods and display the results in Figure 8.

The heuristic method had an average error of 20.91% (SD 24.69%). The SVM and Bi-LSTM methods yielded average error rates of 5.85% (SD 5.45%) and 3.17% (SD 2.75%), respectively. Regarding the SD error, the heuristic method, SVM, and Bi-LSTM had values of 24.69%, 5.45%, and 2.75%, respectively.

### Feedback

We deployed the trained model in a hospital setting to collect subjective and quantitative feedback. Feedback providers included patients with varying degrees of NDDs, their family members, and physicians.

#### Subjective Feedback From Patients and Relatives

The feedback from patients and relatives is presented in Textbox 5.

**Subjective feedback from patients and relatives.**

**Question: “How was your father diagnosed with Parkinson’s?”**

- Answer: “A long time ago, I noticed that my father was a little unsteady in walking. We thought it was a previous lumbar spondylosis attack and didn’t consider neurological diseases. It was too much trouble for the elderly to queue up at the hospital, so we procrastinated. Now, the situation has worsened, and he needs assistance to walk.”

**Question: “How would you rate this new assessment method that we have implemented?”**

- Answer: “This innovative approach only requires me to follow the landmarks and walk a few laps. Previous tests were time-consuming and laborious and I had to wear some equipment that made me uncomfortable. Today’s testing method was mentally relaxing.”

**Question: “Can you share your feeling about this gait assessment method?”**

- Answer: “I feel somewhat self-conscious about the way I walk, but your creation allows me to complete the test quickly, sparing me from enduring a lengthy assessment process.”

**Question: “What do you think is the biggest difference between this assessment and the tests you have done before?”**

- Answer: “Simplicity and convenience. I think the idea you provided is significant; there’s no need to wear devices, and testing is done quickly. Perhaps we can complete these assessments at home in the future. I look forward to your further results.”

**Question: “Do you like the format of our assessment?”**

- Answer: “I like it, I think the report is generated very fast, faster than the ones I did before.”

**Question: “Do you think the results of our equipment are trustworthy?”**

- Answer: “Why not? AI is developing rapidly and taking over human work in many fields. To be honest, I’m more willing to trust your digital devices than the manual scale tests used before.”

#### Subjective Feedback From Physicians

The feedback from physicians is presented in Textbox 6.

**Subjective feedback from physicians.**

**Question: “Do you think our assessment method has advantages over the scale method, and can our method replace the scale method in your opinion?”**

- Answer: “In the past, we often needed to evaluate the patient’s video and manually make the scale. But I feel that the data provided by your assessment method is much more accurate than handcrafted scales. I personally believe that our work would be much less stressful if we could use your results consistently in the future.”

**Question: “Do you think our method has helped you in any substantial way in your work?”**

- Answer: “Making scales requires us to have a lot of communication with patients. This device can improve this situation and avoid the omissions that occur when we evaluate patient parameters ourselves.”

**Question: “Do you think our assessment method provides you with enough information to complete a complementary diagnosis?”**

- Answer: “Of course, it provides a lot of parameters and indicators to consider, which is sufficient for us.”

**Question: “What do you think are the shortcomings of our algorithm during use?”**

- Answer: “When the patient is overdressed or wearing dark clothing all over the body, the results sometimes do not make sense.”

### Quantitative Feedback

The results for each metric are presented in Tables 5 and 6.

**Table 5:** The distribution of quantitative scores collected from 100 participants for our method.

Metric	Score of 1, n (%)	Score of 2, n (%)	Score of 3, n (%)	Score of 4, n (%)	Score of 5, n (%)	Weighted average score
Accuracy (n=7)	0 (0)	0 (0)	0 (0)	1 (14)	6 (86)	4.86
Convenience (n=93)	0 (0)	0 (0)	1 (1)	3 (3)	89 (96)	4.95
Practicability (n=7)	0 (0)	0 (0)	0 (0)	2 (29)	5 (71)	4.71
Invasiveness (n=93)	88 (95)	3 (3)	1 (1)	1 (1)	0 (0)	1.09
Reliability (n=7)	0 (0)	0 (0)	1 (14)	3 (43)	3 (43)	4.29

**Table 6:** The distribution of quantitative scores collected from 100 participants for the conventional scale-based method.

Metric	Score of 1, n (%)	Score of 2, n (%)	Score of 3, n (%)	Score of 4, n (%)	Score of 5, n (%)	Weighted average score
Accuracy (n=7)	1 (14)	3 (43)	2 (29)	1 (14)	0 (0)	2.43
Convenience (n=93)	64 (69)	17 (18)	7 (8)	5 (5)	0 (0)	1.49
Practicability (n=7)	0 (0)	1 (14)	3 (43)	2 (29)	1 (14)	3.43
Invasiveness (n=93)	2 (22)	7 (7)	14 (15)	29 (31)	41 (44)	4.08
Reliability (n=7)	0 (0)	2 (29)	2 (29)	2 (29)	1 (14)	3.29

## Discussion

### Principal Findings

#### Results Comparison

Regarding the classification results (Figures 6 and 7), the Bi-LSTM model outperformed the SVM model in all 3 aspects (average precision, average recall, and average *F*_1_-score). In the parameter calculation aspect, both SVM and Bi-LSTM demonstrated distinct advantages over the heuristic method. Bi-LSTM showcased superior performance with the lowest average error (3.17%, SD 2.75%). The comparative experiments conducted in this section illustrated that (1) the integration of spatiotemporal features, semantic segmentation, and GPM is effective, addressing the weaknesses prevalent in heuristics, and (2) the time-series model yielded more impactful results, with Bi-LSTM exhibiting the best performance.

#### Prediction Rationality

**Figure 9:**
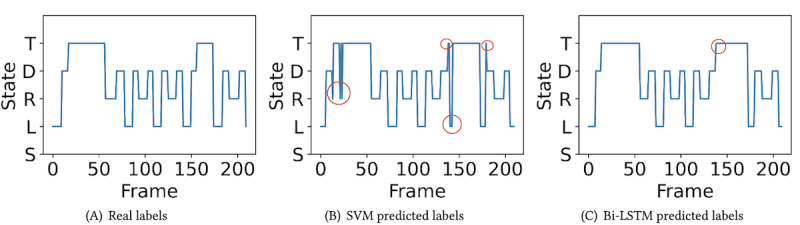
The visual comparison of real labels and predicted gait state labels by support vector machine (SVM) and bidirectional long short-term memory (Bi-LSTM; the circled areas represent the prediction errors). (A) Real labels; (B) SVM predicted labels; (C) Bi-LSTM predicted labels.

The validity of the predictions generated by both the SVM and Bi-LSTM models was evaluated. This was achieved by generating predictions for a given data sample, as shown in Figure 9.

Apparently, the SVM results have many intermittent jumps. In addition, the classification and gait segmentation results show that SVM performs poorly on gait semantic segmentation evaluation (77.5% when tolerance is 2), albeit demonstrating decent performance on precision, recall, and *F*_1_-score on classification (86.99%, 86.62%, and 86.67%, respectively). On the basis of these observations, we offer the following insights.

From the gait semantic perspective, intermittent jumps indicate abrupt transitions between different gait states, which is incongruous with a person’s natural walking pattern. Therefore, the SVM prediction reasonability has significant room for improvement.

The growing discrepancy in semantic location accuracy between SVM and Bi-LSTM underscores that reliance on the 3 common indicators (precision, recall, and *F*_1_-score) can obscure SVM’s substantial weakness in distinguishing gait semantic locations. The number of key semantics is much lower than the total number of frames, implying that, even if key points are inaccurately predicted, the large frame cardinality could compensate for SVM’s accuracy, thus concealing SVM’s errors in predicting gait semantics.

In contrast, Bi-LSTM is very stable on both frame accuracy indexes and key semantic segmentation accuracy. Although the figure shows that the outcome of Bi-LSTM still has a small defect, it will not affect the final parameter calculation as the data in the turning interval will not be covered for the final calculation (introduced in the *Segmentation, GPM, and Calculation* section).

In theory, Bi-LSTM allows the model to integrate information from the preceding and succeeding frames, resulting in a stable model that is resistant to abrupt gait state transitions. Conversely, SVM relies solely on single-frame features without contextual information, causing the prediction result to be susceptible to sudden gait state transitions and a lack of robustness for the state transition of long-term data. In general, in the task of gait assessment, an algorithm based on the Bi-LSTM model is a more appropriate choice.

#### Feedback Analysis

In this section, feedback from patients and their families is denoted by the prefix *P*, whereas physician feedback is indicated by the prefix *D*.

P1 highlighted the difficulty in recognizing early-stage NDDs as they are easily confused with other conditions, which underscores the importance of our research.

P2, P3, P4, and P5 indicated that this study offers an effective way to prevent patient discomfort during gait testing. It boasts high accuracy and rapid testing and encourages a natural gait pattern during testing, thereby ensuring accurate assessment results. P6 underscored the shortcomings of the traditional scale-based tests, suggesting that our method is more reliable from a user perspective. D1 and D2 indicated that our method provides a more efficient and labor-saving assessment strategy that could potentially reduce bias in their judgment. D3 suggested that our method offers comprehensive information, enabling a multifaceted analysis. D4 pointed to an issue with the Kinect’s performance during data collection when patients are heavily or darkly dressed, which limits our model’s performance.

The overall distribution in Tables 5 and 6 demonstrates that our algorithm was generally favorably rated by both patients and physicians, faring better than traditional scale-based approaches. The high convenience (4.95) and reliability (4.29) ratings for our method suggest a low acceptance and lack of confidence in the current scale-based approach. In addition, the rating for invasiveness (1.09 for our method vs 4.08 for the scale-based method) indicates that the scale-based approach may be perceived as intrusive because of the extensive communication required between physicians and patients during the data collection process, potentially infringing on patient privacy. In contrast, our method was considered more respectful of patients’ feelings.

### Comparison With Prior Work

In previous work, although there are also related works that apply the high-end results of digital devices to the evaluation of gait parameters in NDD, these have either focused on using wearable devices [13-15,39] such as sensors to directly obtain various gait parameters of patients or used bulky and expensive optical [40] equipment to complete accurate gait calculations. The low precision and invasiveness of the former and the expense of the latter have become obstacles to “promoting AI for the auxiliary diagnosis and treatment of NDD.” In addition, some heuristics [22-26] that may generalize at scale in hospital scenarios also provide solutions, and we compared our algorithm with the heuristics that perform optimally on this task. The comparison of results shows that our method outperforms the best heuristics by >15% in terms of both the average error and average SD error of the parameters. Taken together, our method has the following advantages over previous methods: (1) we combined composite spatiotemporal features extracted from human lower limbs in motion; (2) we adopted a time series–based deep learning model to make it possible to comprehensively use the semantic information in the context; (3) we relied on precise gait semantics to calculate gait parameters and strictly limited the gait cycles involved in the calculation through the GPM mechanism; (4) we used a single Kinect to complete data collection and accurate prediction, which reduced the cost; and (5) we adopted a noncontact assessment method throughout the process to provide participants with better emotional care and ensure that their behavioral patterns were as consistent as possible with those of the state of nature.

### Limitations

Our study has the following limitations. First, the scale of data used for training and testing the model consisted of 41 samples. Although each sample contained thousands of frames that could be used for training and testing and we tried to make the distribution of the data set as differentiated as possible, it is still challenging to guarantee that it contains all gait patterns. Nonetheless, we can increase the scale of the data set or train an ensemble model for more precise assessments. We are currently collaborating with more hospitals to collect a broader range of gait data. Second, the data noise caused by the limitations of the Kinect itself cannot be overcome by our algorithm. Third, we do not provide direct evidence of the specific value of the improvement in the accuracy of this method compared with the traditional scale-based measurement.

Although we offer precise, uniform gait parameters with controlled errors compared with the traditional scale-based method, the issue lies in the fact that we can only guarantee the accuracy of the parameters, whereas the physician’s assessment of the patient’s symptoms (eg, degree of memory degradation) that are not captured by the parameters remains dependent on the physician’s own standard. Therefore, this study aimed to assist physicians in their decision-making by eliminating as many measurement errors as possible rather than provide a fully automated, manual alternative to diagnosis.

In addition, the data set is imbalanced regarding sex (30/41, 73% male participants vs 11/41, 27% female participants), which might affect the model’s performance. Moreover, we have not yet explored the potential correlations between age and sex and the input parameters of the classifiers. This can be explored as a future research direction.

### Conclusions

Gait assessment results as a crucial indicator of NDD can aid physicians in providing a more accurate early disease diagnosis. However, existing methods often present various drawbacks: traditional scales are time-consuming and prone to high error rates; contact assessments are invasive; optical instruments, though accurate, are costly; and heuristic methods, although simple and straightforward, fail to meet clinical requirements in terms of accuracy. Consequently, we proposed a contactless gait assessment method that leverages the advanced results of deep learning, combines rich spatiotemporal features, and incorporates timing information from data to achieve a highly accurate gait assessment (with an average error of only 3.17% [SD 2.75%] and a 15% reduction in the error margin compared with previous heuristic optimal methods). This study shows the feasibility and rationality of using deep learning to assist physicians in the early diagnosis of NDDs and targeted rehabilitation. Our method can serve as a diagnostic support system, benefiting physicians and patients alike through its low equipment costs, portable deployment, accurate results, and minimal invasiveness.

Despite the promising results, our study has some limitations. First, the sample size in our study may not be large enough to generalize our findings to the broader population of patients with NDDs. Second, the algorithm may not perform as well when dealing with patients wearing dark or bulky clothing, which may affect the accuracy of the Kinect-based data collection.

In future research, we plan to explore the application of our method in a broader range of NDDs while promoting it in more hospitals to provide convenience for patients and physicians. In addition, we aim to investigate how to integrate our approach with other diagnostic tools to provide a more comprehensive assessment, thereby further enhancing the early diagnosis and management of NDDs.

## Abbreviations

Bi-LSTM: bidirectional long short-term memory
GPM: gait pair mechanism
NDD: neurodegenerative disease
SVM: support vector machine

## Data Availability

The data sets generated and analyzed in this study were provided by the Institute of Software, Chinese Academy of Sciences, and are not publicly available because of data protection requirements. Access to the data is restricted; however, researchers who are interested in the data may request access by contacting the corresponding author (WS). Requests for data access will be considered on a case-by-case basis subject to the data provider’s approval and any applicable data protection regulations.
